# Visual discrimination and resolution in freshwater stingrays (*Potamotrygon motoro*)

**DOI:** 10.1007/s00359-020-01454-2

**Published:** 2020-12-02

**Authors:** Martha M. M. Daniel, Laura Alvermann, Imke Böök, Vera Schluessel

**Affiliations:** Institute of Zoology, Rheinische Friedrich-Wilhelms-Universität Bonn, Poppelsdorfer Schloss, Meckenheimer Allee 169, 53115 Bonn, Germany

**Keywords:** Behavioral cognition, Visual acuity, Shape, Learning, Elasmobranch

## Abstract

*Potamotrygon motoro* has been shown to use vision to orient in a laboratory setting and has been successfully trained in cognitive behavioral studies using visual stimuli. This study explores *P. motoro*’s visual discrimination abilities in the context of two-alternative forced-choice experiments, with a focus on shape and contrast, stimulus orientation, and visual resolution. Results support that stingrays are able to discriminate stimulus-presence and -absence, overall stimulus contrasts, two forms, horizontal from vertical stimulus orientations, and different colors that also vary in brightness. Stingrays tested in visual resolution experiments demonstrated a range of visual acuities from < 0.13 to 0.23 cpd under the given experimental conditions. Additionally, this report includes the first evidence for memory retention in this species.

## Introduction

*Potamotrygon motoro* has a distribution that overlaps that of other Potamotrygonid rays, such as *Potamotrygon wallacei*, *Paratrygon aiereba* (Oliveira et al. 2017), and *Potamotrygon falkneri* (Garrone Neto and Uieda 2012). While it is unclear how reliant *P. motoro* is on visual information in its natural environment, the marked differences between species skin patterns (Rosa 1985) raise the question of whether these play a role in conspecific discrimination and which aspects of the patterns could be important in such a case. It has been shown that *Potamotrygon motoro* uses vision to orient spatially (Schluessel and Bleckmann 2005; Schluessel et al. 2015; Schluessel and Ober 2018), and the species has been successfully trained in cognitive behavioral studies using visual stimuli (e.g. Seifert 2017, Daniel and Schluessel 2020). In the field, juveniles have furthermore been documented preying on snails above water level during the day (Garrone-Neto and Sazima 2009), a behavior which likely requires visual perception.

*Potamotrygon motoro* can discriminate colors (Seifert 2017) and process numerical information (Christofzik 2016; Niederbremer 2019), but its abilities to distinguish geometric forms, judge stimulus orientation, and register different levels of detail have previously remained unexplored. Christofzik (2016) and Niederbremer (2019) conducted experiments on quantity discrimination with stimuli that incorporated various groups of circles, triangles, and squares of different contrasts. Successful discrimination implied that stimuli were perceived as consisting of multiple separate entities, but it remained ambiguous whether this perception was based on elements of form or contrast. In a serial reversal learning experiment, Daniel and Schluessel (2020) used stimuli that differed in orientation, brightness, and color, but it was not tested which of these properties the stingrays used for discrimination. The present study thus sought to clarify and elaborate on previous experiments with this species while providing leads for further research. Similar discrimination experiments conducted on the grey bamboo shark (*Chiloscyllium griseum*) showed that sharks could easily discriminate between forms and objects, based on shape and contrast but not color (Fuss et al. 2014; Schluessel et al. 2014; Schluessel 2015).

Chondrichthyan eyes are much more diverse than teleost eyes, with respect to pupil shape, size, and constriction/dilation rate, as well as lens shape and capacity for accommodation, so the elasmobranch eye cannot be successfully summarized in a ‘typical fish-eye model’ like that of teleosts (Sivak 1991). *Potamotrygon motoro* has a multifocal lens, which, when optimally positioned, should allow for compensation of the chromatic aberration experienced by other lens types (Gustafsson et al. 2012). Unfortunately, lens positioning remains undescribed in this species because the eye was too small to reliably determine the lens suspension mechanism via dissection (Gustafsson et al. 2012). It was however observed that part of the iris overlays a section of the lens periphery (Gustafsson et al. 2012).

**Fig. 1:**
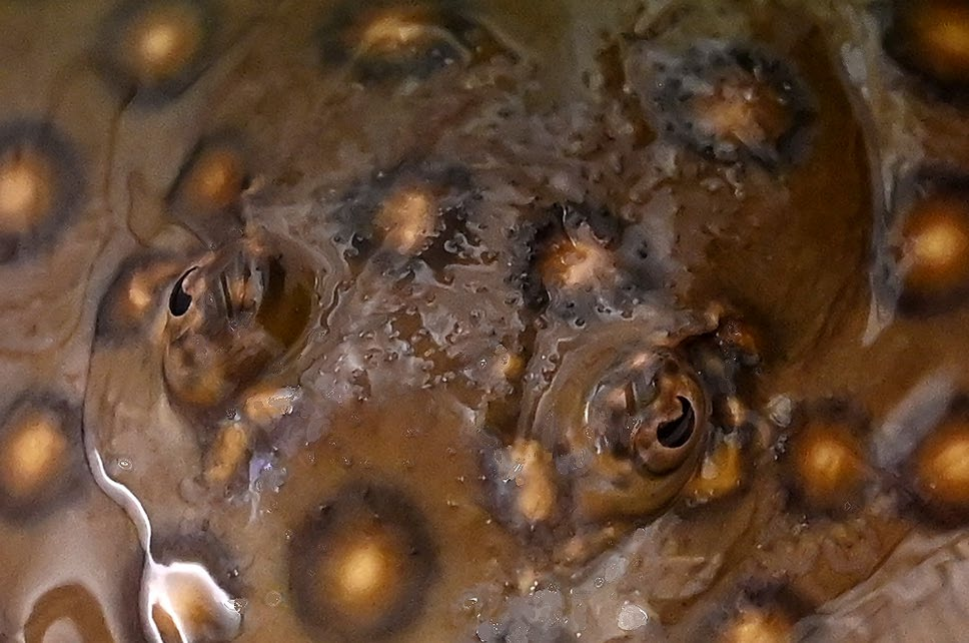
Eye of *Potamotrygon motoro* at 320 lx, displaying crescent-shaped pupil. The depicted individual has buried its body in the sand (picture by S. Büttner)

The retina of *P. motoro* has a visual cell layer that is comprised of rod-shaped and cone-shaped cells in a ratio of 6–7:1, which is low by comparison to other elasmobranchs (Ali and Anctil 1974) and may be an indication that photopic vision is important to the species (Schluessel and Bleckmann 2005). Several batoids are both physiologically (Bedore et al. 2013; Hart et al. 2004; Theiss et al. 2007) and behaviourally (Van-Eyk et al. 2011) able to perceive and discriminate colour, using multiple cone visual photopigments. This together with *Potamotrygon motoro*’s ability to discriminate colors (Seifert 2017) could demonstrate that photoreceptors in elasmobranchs function similarly to the rods and cones in teleost and mammal retinae. However, rays are among those vertebrates that possess crescent-shaped pupils (Fig. 1), formed by an expanding and contracting pupillary operculum, and this poses consequences for perception, including for visual resolution (Murphy and Howland 1991).

This study is the first approach to several visual discrimination experiments with *Potamotrygon motoro* and includes the first exploration of resolution in the species. It additionally reports the first evidence for memory retention of a visual discrimination task in *P. motoro.* The hypotheses tested in visual discrimination experiments were that *P. motoro* would be able to discriminate between (A) stimulus-presence and stimulus-absence, (B) different forms, (C) different overall stimulus contrasts, and (D) horizontal and vertical stimulus orientations. Since differences in peripheral sensory input have been documented between the sexes in the blue-spotted fantail stingray (*Taeniura lymma*) (Kempster et al. 2013), we also included a comparison of how long it took males and females to achieve learning criterion in one of the stimulus-presence vs. stimulus-absence experimental groups. A further comparison was included for stingray diameter.

## Materials and methods

### Animals and maintenance

**Fig. 2:**
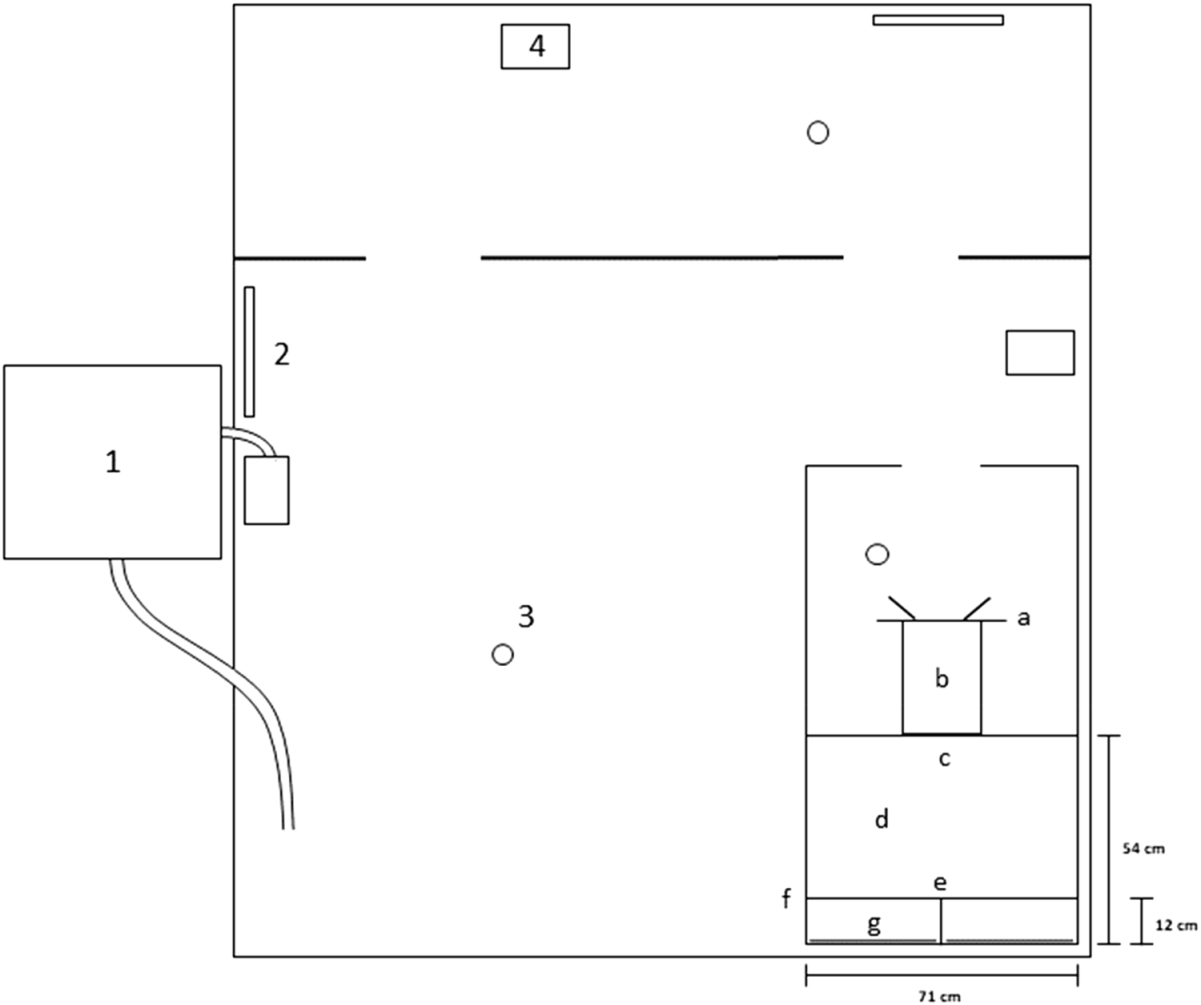
Holding tank (2.30 m × 2.07 m × 0.40 m) with (1) filter system, (2) aquarium heaters, (3) air stones, and (4) pumps and incorporating the experimental apparatus: (a) guillotine door 1, (b) starting box, (c) guillotine door 2, (d) decision area, (e) clear barrier, (f) decision line, (g) stimulus wall (adapted from Daniel and Schluessel 2020)

Experiments were conducted at the Institute of Zoology at Bonn University on eighteen juvenile and sub-adult *Potamotrygon motoro* individuals in three separate groups. One group was comprised of four males and one female, aged 2–3 months and ranging from 12 to 15 cm in diameter. These were naive to experiments and on loan from Frankfurt Zoo. Another was comprised of four females and four males, aged 9–10 months and ranging from 8 to 14 cm in diameter. These were also naive to experiments and on loan from Antwerp Zoo. One of the Antwerp individuals dropped out of the experiments following the stimulus-presence vs. stimulus-absence experiment. The third group contained three males and two females, aged 3–4.5 years and ranging in diameter from 22 to 30 cm. These were not naïve to experiments and were on loan from Frankfurt Zoo. Each group of stingrays was maintained in 1300 L of water in a 2.30-m × 2.07-m × 0.40-m holding tank that contained an experimental apparatus (Fig. 2), which was freely open to the animals outside of experimental sessions. Temperature was maintained between 27 and 29 °C with Eheim Jäger 3618 Aquarium Heaters, conductivity was kept between 350 and 420 μS using Aqua Medic AB Reef Salt, hardness was kept at a KH value of 3 with Reef Life System Coral B buffer, and water was constantly circulated with two pumps, one Aqua Medic ECO Runner 2700 pump and one Pontec PondoVario 1000 pump. Three air stones for oxygenation also contributed to water mixing. Water was filtered at a constant rate of 270 L/h and exchanged at least once per week to keep nitrite values below 0.05 mg/L. Stingrays were fed a diet of 70% shrimp and 30% earthworms. Light intensity in the experimental room was 320 lx.

### General methods

Testing always consisted of two-alternative forced-choice experiments, in which one of the stimuli was associated with a food reward while the other was not. Two daily sessions were conducted six days per week, with ten trials per session. Animals acclimated to their surroundings by swimming freely around the experimental tank. They were then trained to swim through the first guillotine door of the experimental apparatus, into the starting box, and through the second guillotine door to cross the decision area and search for food along the stimulus wall. Stimuli (Table 1) were presented in pseudo-random order, appearing equally often on either side of the barrier per session but never on the same side more than twice consecutively.

**Table 1:**
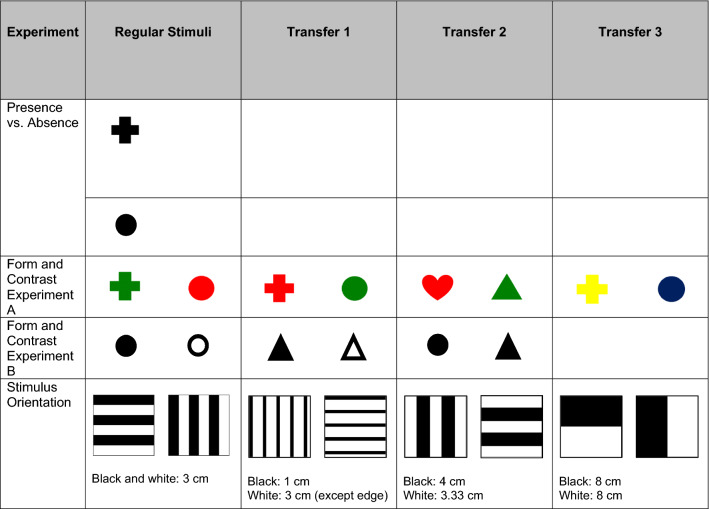
Stimuli used in visual discrimination experiments

All stimuli were printed on a standard white background and laminated. Stimuli in the first presence vs. absence discrimination and in the first form discrimination were printed on paper 15 cm × 21 cm large. The shapes in these experimental sets each had a diameter of 8 cm. Each stimulus in the second experimental group for presence vs. absence discrimination as well as in the second group for form discrimination was printed on standard A4 paper. The shapes presented in these experimental sets each had a total area of 59.86 cm^2^. Stimuli used in orientation discrimination experiments each had a total area of 18 cm^2^. Transfer stimuli differed from the regular stimuli in number and thickness of stripes, and stripe thickness is reported for both contrasts under the depicted stimuli

Timing started as soon as the tip of an animal’s disk passed the threshold of the second guillotine door and ended as soon as the tip of its disk passed the decision line. If the subject chose the side with the form designated for reward-association, food was rewarded as soon as the decision line was crossed. If a subject chose the stimulus not designated for reward-association, no food was rewarded. In the latter case, the animal was guided back to the starting box after approximately 5–10 s, if it did not return on its own. A correct choice granted enough time for consumption of the reward.

Throughout the experiments in this study, learning was evaluated in terms of sessions to criterion (e.g. Schluessel and Bleckmann 2005; Leal and Powell 2012; Fuss et al. 2014; Lucon-Xiccato and Bisazza 2014). When the learning criterion (LC) of ≥ 70% correct choice in three consecutive sessions was achieved, the task was considered complete and a transfer test phase followed (e.g. Schluessel and Bleckmann 2005; Fuss et al. 2014), where applicable. The random presentation of unfamiliar, unrewarded stimuli in transfer tests asks an animal to divulge the discrimination strategy it uses during regular trials, in that the resulting choices are indicative of what stimulus properties the animal is attuned to. Once LC was achieved and transfer testing commenced, sessions were supposed to be continued at an 80% reward scheme to prevent the animals from immediately associating unfamiliar stimuli with a lack of reward (e.g. Schluessel et al. 2015; Schluessel and Ober 2018). This reduced scheme was however followed by a drop in regular performance, so a 90% scheme was implemented instead.

All data analyses in the reported study were conducted with R statistical software, versions 3.5.2 and 3.5.3. The group results for each transfer test were analyzed using a Generalized Linear Mixed Model with Laplace Approximation, as recommended by Garamszegi (2015), and transfer trial times and their corresponding average session times were compared with the Wilcoxon signed-rank test, following testing with the Shapiro–Wilk normality test. Individual stingrays were evaluated for overall stimulus choices in each transfer test using Exact Binomial tests. While not optimal, binomial tests were the most appropriate statistics available for individual analyses. It is necessary to note that the results of each trial cannot be seen as independent from other trials within a session, and experience from prior trials in any session can inform an animal’s subsequent decisions.

### Visual discrimination

The first part of this study tested the abilities of *Potamotrygon motoro* to visually discriminate stimulus-presence vs. -absence, form, contrast, and stimulus orientation. The regular stimuli used for each experiment are presented in Table 1.

#### Stimulus-presence vs. stimulus-absence

To make sure that naive animals were not physically or cognitively impaired, it was first tested whether they could discriminate between a symbol and a blank white card (Table 1). Thirteen stingrays were presented with a blank white stimulus versus a black form, either a cross (*n* = 5, Table 1) or a circle (*n* = 8, Table 1) on a white background. Average and median numbers of sessions to LC were calculated for all thirteen stingrays, while separate averages and medians were also calculated for the two groups. For the eight stingrays presented with a circle, two-sample *t* tests were used to compare the numbers of sessions to LC between sexes and between stingrays 12–14 cm in diameter vs. stingrays 8–10 cm in diameter.

#### Form, color, and contrast

Two different approaches to test form discrimination were applied. In Experiment A, the five animals that had been trained to approach a cross instead of a blank white card were tested using a stimulus pair that consisted of two different shapes with equivalent diameters (i.e. cross vs. circle, Table 1). Shapes were first both presented in black, but as stingrays did not achieve LC within 30 sessions, shapes were later presented in colors which also differed in brightness (i.e. green cross vs. red circle, Table 1). Following achievement of LC, three sets of transfer stimuli were presented randomly to determine whether the successful discrimination of colorful shapes was based on color/brightness, form, or both (Table 1). Transfer 1 stimuli were designed to test whether animals paid more attention to familiar color/brightness or familiar form, and Transfer 2 stimuli were designed to test whether stingrays could discriminate based on color/brightness when forms were unfamiliar. Transfer 3 stimuli were designed to test whether stingrays could discriminate based on shape when color/brightness was unfamiliar. No more than three transfer trials were conducted per session, with each session still consisting of ten regular trials. Fourteen total trials for each transfer stimulus pair were conducted per animal.

In Experiment B, seven of the animals that had been previously trained to approach a circle were presented with a regular stimulus pair that did not differ in shape (i.e. black circle vs. outlined circle, Table 1) but differed in ratios of black to white and overall brightness, hereafter referred to together as contrast. Following achievement of LC, two sets of unrewarded transfer stimuli (Table 1) were presented randomly with no more than three transfer trials per session, for a goal of at least 20 total trials for each transfer stimulus pair per animal. If a session included three transfer trials, the number of regular trials was raised from 10 to 14. Transfer 1 stimuli imitated the stimuli of regular trials with regard to contrast and tested whether stingrays had in fact used contrast in the discrimination process and were capable of applying this knowledge to a similar but new task. Transfer 2 tested whether stingrays still chose the ‘correct’ stimulus from regular trials when it was paired with a new alternative stimulus of different form, featuring the same color and area. The use of these two transfer sets elucidated whether rays had based their original choices solely on contrast differences or if they had also paid attention to general form.

Normally, transfer tests would not have been conducted until performance remained consistently above 70% correct choice, even though LC had been achieved. However, the animals in Experiment B did not maintain consistent performances for more than four consecutive sessions at a time. For these stingrays, it was therefore decided that a transfer test would be conducted in a given session if the stingray chose correctly in the first four to five trials, but if performance decreased later in the session, no further transfer tests would be conducted in that session. Due to the inconsistent performances of stingrays, a different number of transfer trials was conducted with each individual that participated in transfer testing (16 tests at minimum, *n* = 3). The last 16 transfer tests of each animal were included in analysis.

### Stimulus orientation

The ability of stingrays to discriminate stimulus orientation was investigated using a stimulus pair of horizontally (positive stimulus) and vertically (alternative stimulus) oriented black and white stripes (Table 1). Following achievement of LC, a transfer test phase was conducted to determine whether orientation or overall stimulus image had been used in discrimination. Three sets of unrewarded transfer stimuli (Table 1), varying in line width and number of lines, were presented randomly among 12 regular trials per session, with no more than two transfer trials per session. Twenty total trials were conducted for each transfer stimulus pair per animal.

### Visual resolution

To explore the resolution of *Potamotrygon motoro*, a transfer test phase was conducted in which regular trials still presented stingrays with the striped stimuli from the orientation experiments (Table 1). Eight sets of unrewarded transfer stimuli, in which stripe widths varied from 1 to 10 mm (Table 2), were presented randomly among 12 regular trials per session, with no more than two transfer trials per session. Twenty total trials were conducted for each transfer stimulus pair per animal.

**Table 2:** Widths of stripes (mm) in stimuli used to test visual resolution

Regular	Transfer 1	Transfer 2	Transfer 3	Transfer 4	Transfer 5	Transfer 6	Transfer 7	Transfer 8
30.0	1.0	2.0	10.0	5.0	7.5	6.5	5.5	8.0

Regular stimuli consisted of the same stimuli used to test visual discrimination of stimulus orientation. Each stimulus had a total area of 18 ${\mathrm{cm}}^{2}$. A stimulus pair always consisted of a vertical and horizontal orientation of the same stimulus pattern. Black and white stripes had equal widths in each stimulus

For this experiment, the distance between guillotine door 2 and the decision line was 41.5 cm and the distance from the decision line to the stimulus wall was 11.5 cm (different from Fig. 1). These are the measurements that were used for calculations of visual acuity.

Visual acuity was calculated according to the formula from Parker et al. (2017), as adapted from Nakumara (1968):

1 $${\text{SF}} = \frac{1}{{\left( {2\tan^{ - 1} \left( {\frac{0.5CW}{D}} \right) } \right)\left( {\frac{180}{\pi }} \right)}}$$

where SF stands for ‘Spatial Frequency’, CW describes cycle width (i.e. the width of one adjacent pair of black and white stripes) in mm, and *D* refers to the minimum reaction distance [i.e. in this case, the distance of the decision line from the stimulus wall (115 mm) plus the distance from an animal’s eyes to the edge of its body disk (30 mm)]. The value for CW is the smallest width that each stingray could discriminate with statistical significance during transfer tests.

### Preliminary tests of memory

An additional pilot investigation into the memory capacity of *Potamotrygon motoro* was conducted, in which a 14-day break in reinforcement was included during two of the visual discrimination experiments described above.

In the stimulus-presence vs. stimulus-absence experiments, four stingrays trained with the circle stimulus were given a 14-day break after they achieved LC to see if they could achieve LC again thereafter. A fifth stingray was given a break one session before achieving LC to determine whether the fish had learned the association before achieving three consecutive sessions with ≥ 70% correct choice. If a fish did not achieve LC again within ten sessions after the break, the experiment was discontinued and considered completed. In the case of the fifth individual, which scored ≥ 70% correct choice in the first session after the break, thereby officially achieving LC for the first time, a total of three sessions was conducted after the break to determine whether this performance would remain consistent.

During the training period of the form and contrast Experiment B, three individuals of the group that was trained with contrasting circle stimuli were presented with a 14-day break as well. One of these was given the break after having achieved LC to preliminarily indicate whether memory can be demonstrated with a different stimulus set. Two other stingrays were given the break early in training, after sessions 7 and 14, respectively, to see how quickly LC would be achieved thereafter and whether any potential insights into the learning process might be gleaned from such an approach.

The results of this investigation into memory are interpreted with caution and are reported with the intention of encouraging future research specifically dedicated to the topic of memory.

## Results

**Fig. 3:**
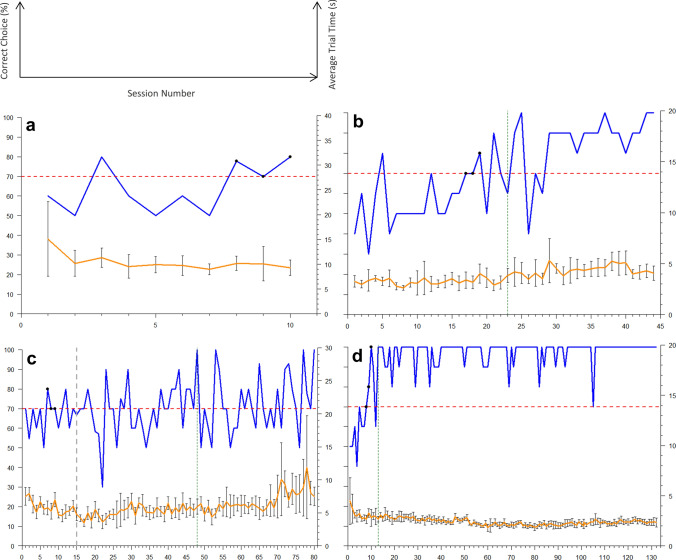
Representative individual learning curves for **a** stimulus-presence vs. stimulus-absence; **b** Form, Color, and Contrast Experiment A (green cross vs. red circle), **c** Form, Color, and Contrast Experiment B (contrasting circles); and **d** Horizontal vs. Vertical. Percent correct choice is shown in blue and average trial time (s) per session is in orange, with error bars indicating standard deviation. Dashed red lines represent the 70% correct choice threshold. Black points indicate achievement of LC. In **b**, **c**, and **d** the dashed dark-green line indicates the session after which transfer testing was commenced. In **c**, the dashed grey line indicates a 14-day break to test memory between sessions 15 and 16

Representative individual learning curves for each experimental phase are displayed in Fig. 3.

### Visual discrimination

#### Presence vs. absence

**Fig. 4:**
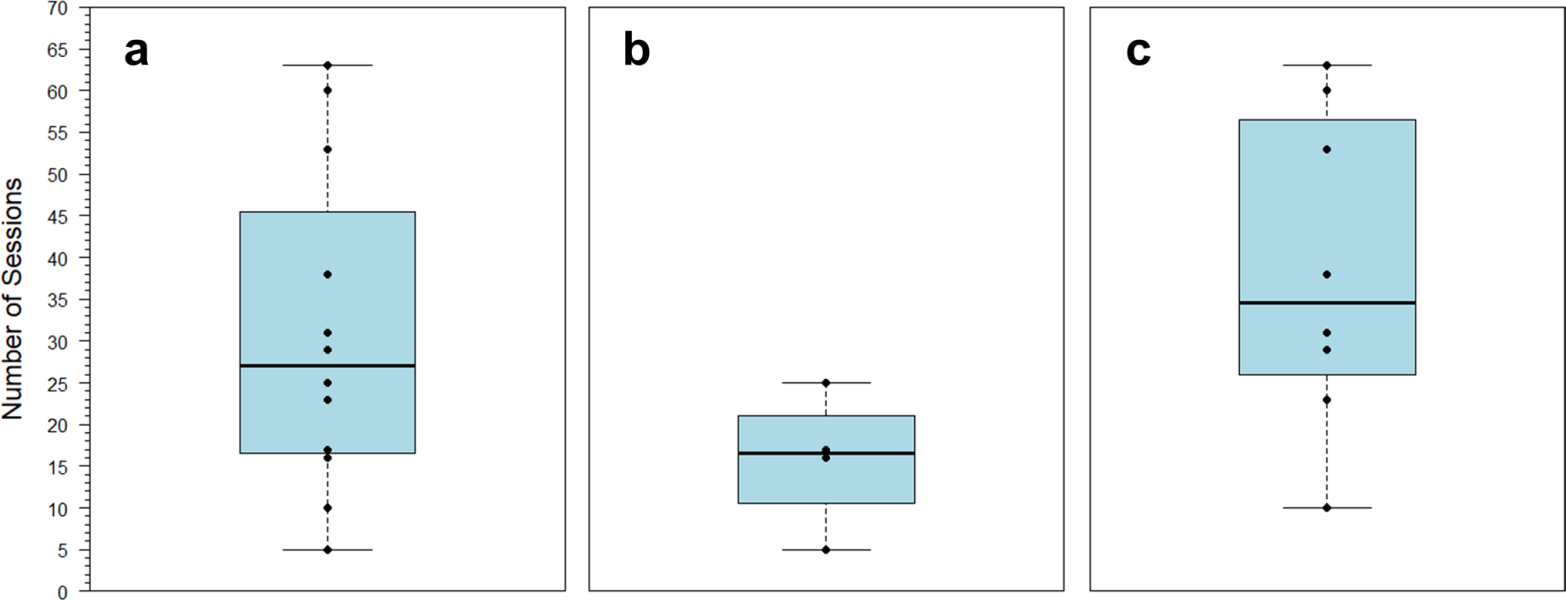
Summary of number of sessions to LC for **a** all 12 stingrays presented with a black form vs. blank white stimulus, **b** subset of four stingrays presented with the cross, and **c** subset of eight stingrays presented with the circle. Bold lines indicate medians, the color-shaded regions represent interquartile ranges (IQRs), and bars indicate ranges to ± 1.5 × IQR. Points indicate individual stingray scores

All stingrays together achieved LC with an average of 31 ± 19 sessions and a median of 27 (Fig. 4, *n* = 12). Average trial time usually decreased across sessions, with the overall mean ranging from 3.48 ± 0.96 s to 17.03 ± 7.99 s between animals. The four stingrays presented with a cross achieved LC with an average of 16 ± 8 sessions and a median of 17 sessions (Fig. 4). Average trial time for this subset usually decreased across sessions for individuals, with the overall mean ranging from 3.48 ± 0.96 s to 8.50 ± 2.11 s between animals (*n* = 4). The eight stingrays presented with a circle achieved LC with an average of 38 ± 19 sessions and a median of 35 sessions (Fig. 4, *n* = 8). Average trial time again usually decreased across sessions for individuals, with the overall mean ranging from 4.59 ± 2.30 s to 17.03 ± 7.99 s between animals (*n* = 8).

**Fig. 5:**
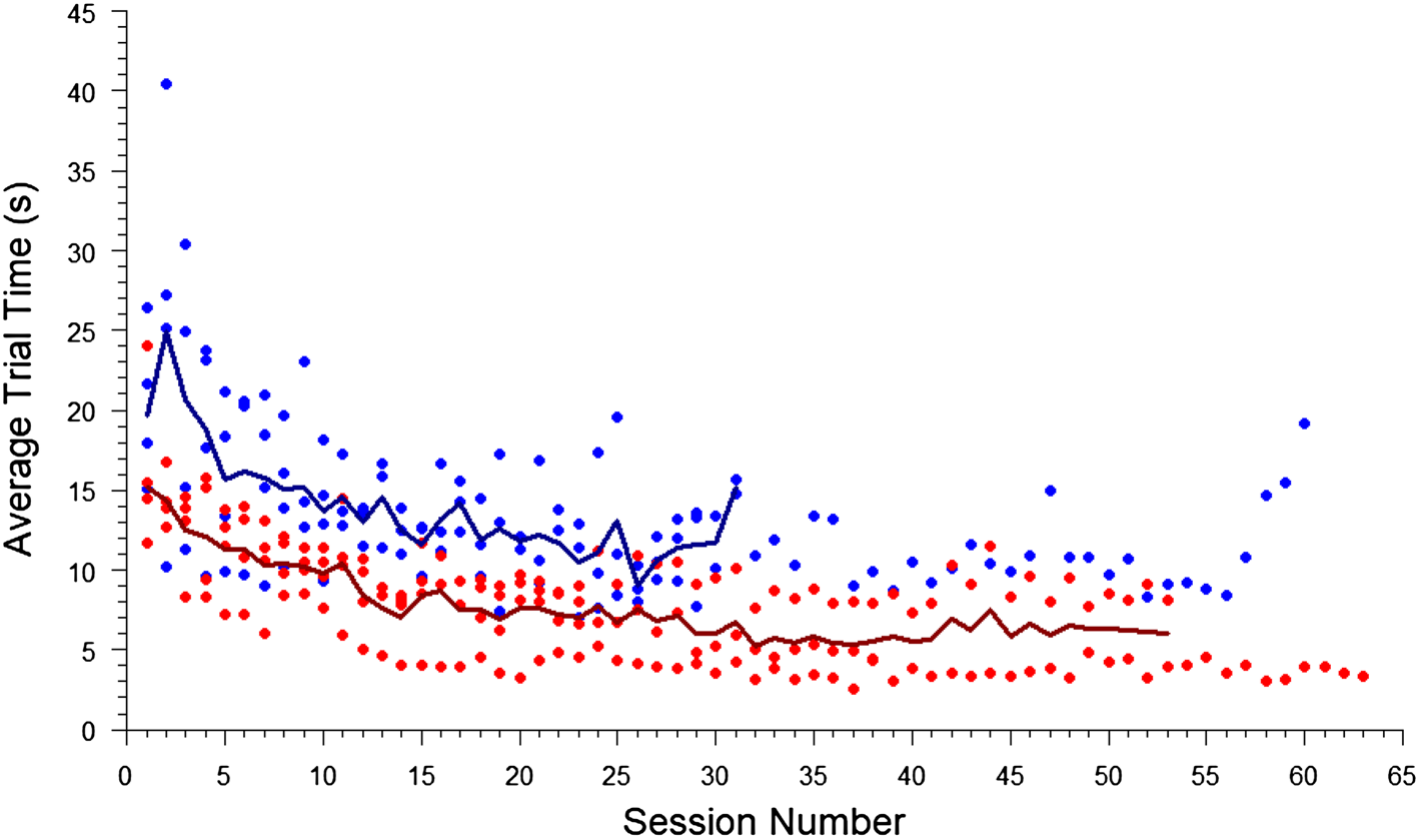
Average trial time (s) for four male (blue) and four female (red) *Potamotrygon motoro*. Lines indicate the average by sex for all sessions in which trials from more than one individual are available. Plotted points show the average trial times of individual stingrays and are an illustration of data spread

For the eight stingrays presented with the circle, there was no significant difference in sessions to LC between stingrays 12–14 cm in diameter and stingrays 8–10 cm in diameter (two-sample *t* test: *t* = 0.087149, *df* = 6, *p* = 0.93). There was also no significant difference in sessions to LC between males and females (two-sample *t* test: *t* = 0.86863, *df* = 6, *p* = 0.42). The potential difference between sexes in average trial time across sessions (Fig. 5) would have been interesting to analyze further given a larger sample size.

#### Form, color, and contrast

**Fig. 6:**
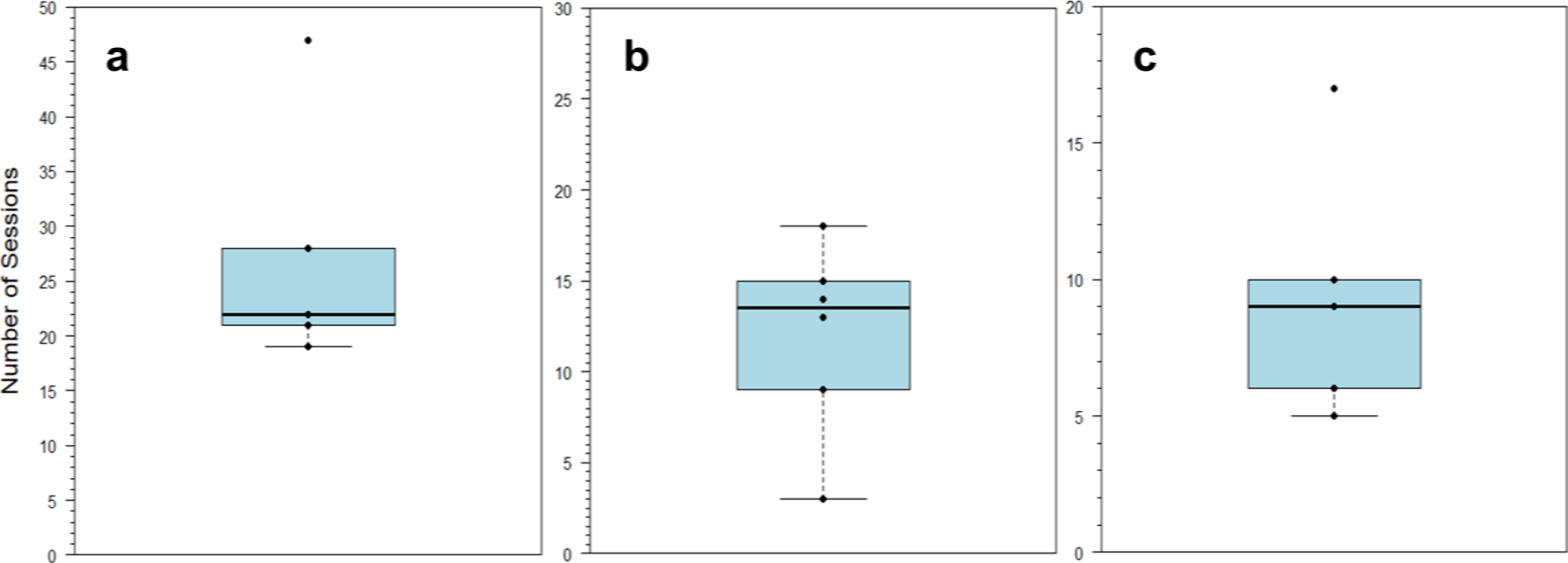
Summary of number of sessions to LC for five stingrays presented **a** with circle vs. cross stimuli in color; **b** with contrasting circle stimuli; and **c** with horizontal vs. vertical stimuli. The bold line indicates the median, the color-shaded region represents the interquartile range (IQR), and bars indicate the range to ± 1.5 × IQR. Points indicate individual stingray scores

In Experiment A, no stingray achieved LC within 30 sessions when presented with a black circle versus a black cross (*n* = 4). These shapes were then presented in color (green cross vs. red circle, Table 1), and stingrays achieved LC with a group mean of 27 ± 11 sessions and a median of 22 sessions (Fig. 6a, *n* = 5).

**Fig. 7:**
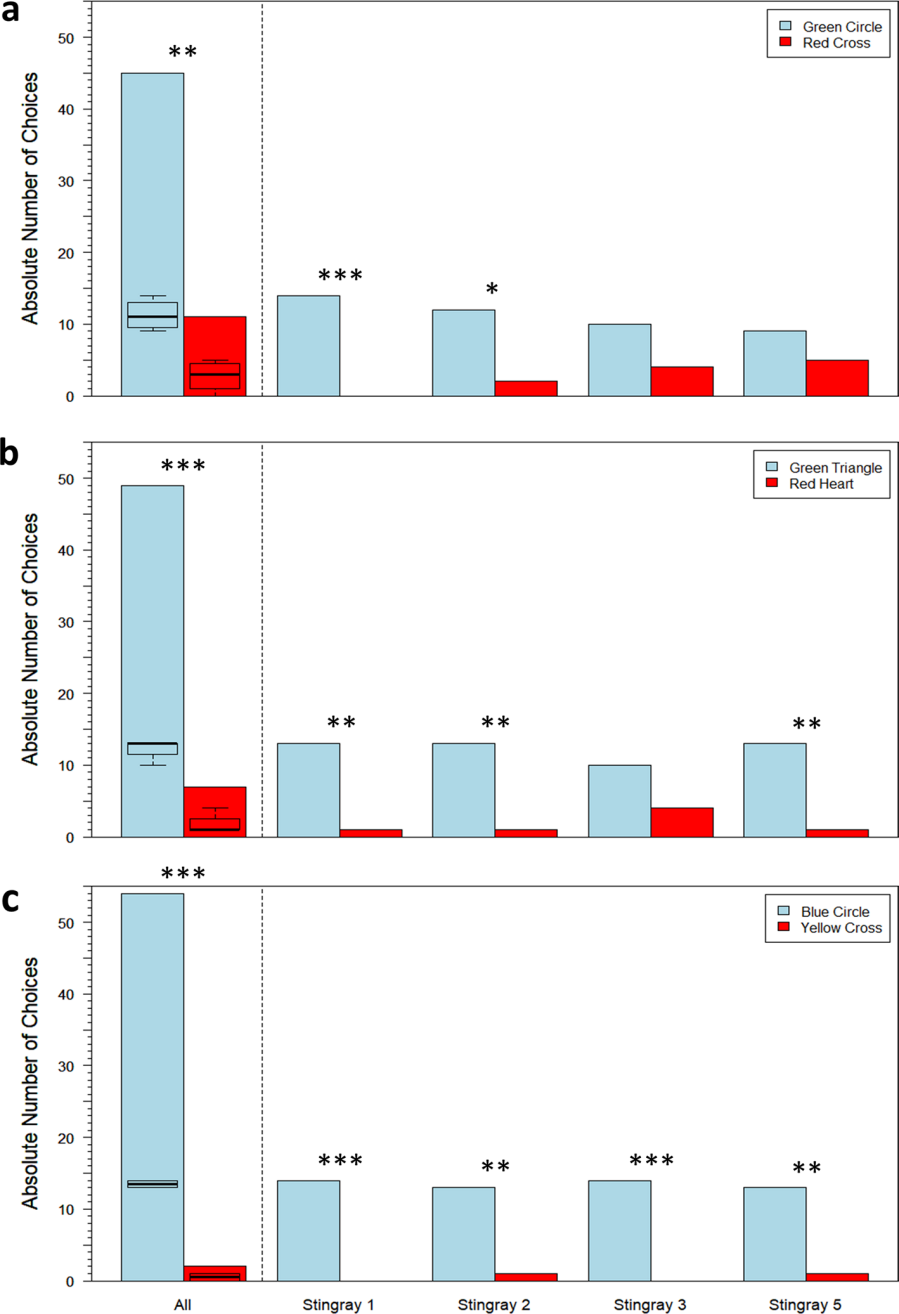
**a** Transfer 1: absolute number of choices between green circle and red cross stimuli for all four individuals compiled, as well as per individual; **b** Transfer 2: absolute number of choices between green triangle and red heart stimuli for all four individuals compiled, as well as per individual; **c** Transfer 3: absolute number of choices between blue circle and yellow cross stimuli for all four individuals compiled, as well as per individual. Boxplots display summary statistics for each stimulus, wherein bold lines indicate medians, color-shaded regions represent interquartile ranges (IQR), and dashed bars indicate ranges to ± 1.5 × IQR. *p value < 0.05; **p value < 0.01; ***p value < 0.001

In Transfer 1, stingrays together significantly often chose the green circle over the red cross (*n* = 4, GLMM: *df* = 54, *z* = 2.972, one-sided *p* value = 0.003, Fig. 7), which shows that color/brightness was chosen over form. In Transfer 2, stingrays together significantly often chose the green triangle over the red heart (*n* = 4, GLMM: *df* = 54, *z* = 4.907, one-sided *p* value < 0.0001, Fig. 7), which shows that color was still used to discriminate. In Transfer 3, stingrays together significantly often chose the blue circle more often than the yellow cross (*n* = 4, GLMM: *df* = 54, *z* = − 4.577, one-sided *p* value < 0.0001, Fig. 7), which indicates that color was still used to discriminate. Individual results for all three transfer tests are also displayed in Fig. 7, with statistical significance determined by the Exact Binomial test (test proportion = 0.5). There was a significant difference between average session time and transfer test time in Transfer 1 (Wilcoxon signed-rank test: *V* = 512, *p* value = 0.02) but not in Transfer 2 (Wilcoxon signed-rank test: *V* = 555, *p* value = 0.11) or Transfer 3 (Wilcoxon signed-rank test: *V* = 966, *p* value = 0.17).

In Experiment B, six out of the seven surviving stingrays that were presented with the contrasting circle stimuli achieved LC, with a group mean of 12 ± 5 sessions and a median of 14 sessions (Fig. 6b). Three stingrays participated in transfer tests. Inconsistent stingray performances resulted in a different number of transfer trials being conducted for each of the individuals. Performance fluctuation also resulted in gaps between the earliest transfer trials for Stingrays 1 and 3, so to avoid the impacts of potential confusion in early transfer trials, the last 16 trials of each animal were used for analysis.

**Fig. 8:**
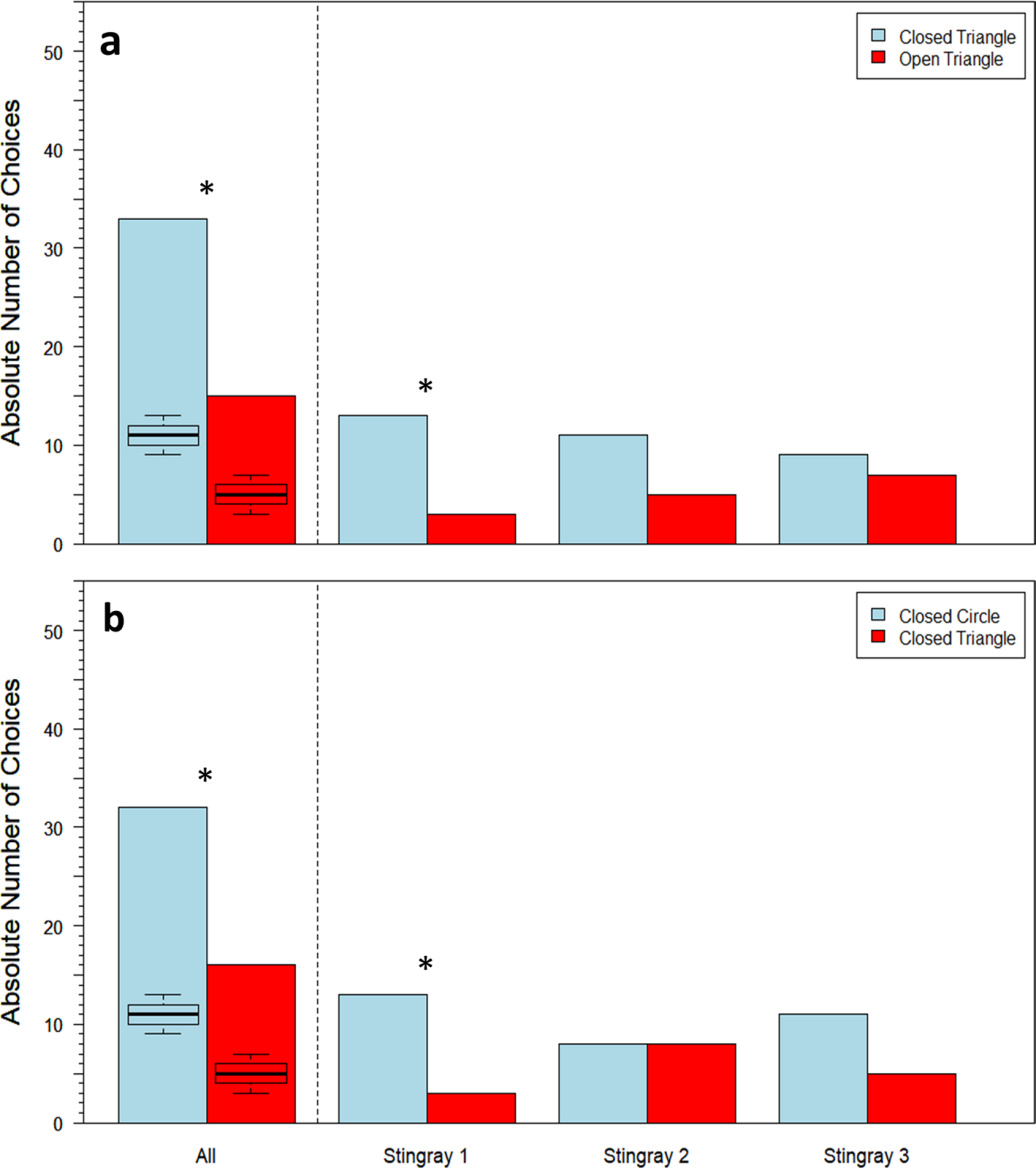
**a** Transfer 1: absolute number of choices between closed triangle and open triangle stimuli in the first 16 transfer tests for all three individuals compiled, as well as per individual; **b** Transfer 2: absolute number of choices between closed circle vs. closed triangle stimuli in the last 16 transfer tests for all three individuals compiled, as well as per individual. Boxplots display summary statistics for each stimulus, wherein bold lines indicate medians, color-shaded regions represent interquartile ranges (IQR), and dashed bars indicate ranges to ± 1.5 × IQR. *p value < 0.05

In Transfer 1, stingrays altogether chose the closed triangle stimulus significantly more often than the open triangle stimulus (GLMM: *df* = 46, *z* = 2.532, one-sided *p* value = 0.01; Fig. 8), showing that discrimination was based on contrast. In Transfer 2, stingrays altogether chose the closed circle stimulus significantly more often than the closed triangle stimulus (GLMM: *df* = 46, *z* = 2.069, one-sided *p* value = 0.04; Fig. 8), indicating that discrimination was based on shape. Individual results for both transfer tests are also displayed in Fig. 8, with statistical significance determined by the Exact Binomial test (test proportion = 0.5). There was a significant difference between average session time and transfer test time in Transfer 2 (Wilcoxon signed-rank test: *V* = 368, *p* value = 0.04) but not in Transfer 1 (Wilcoxon signed-rank test: *V* = 576, *p* value = 0.91).

#### Stimulus orientation

**Fig. 9:**
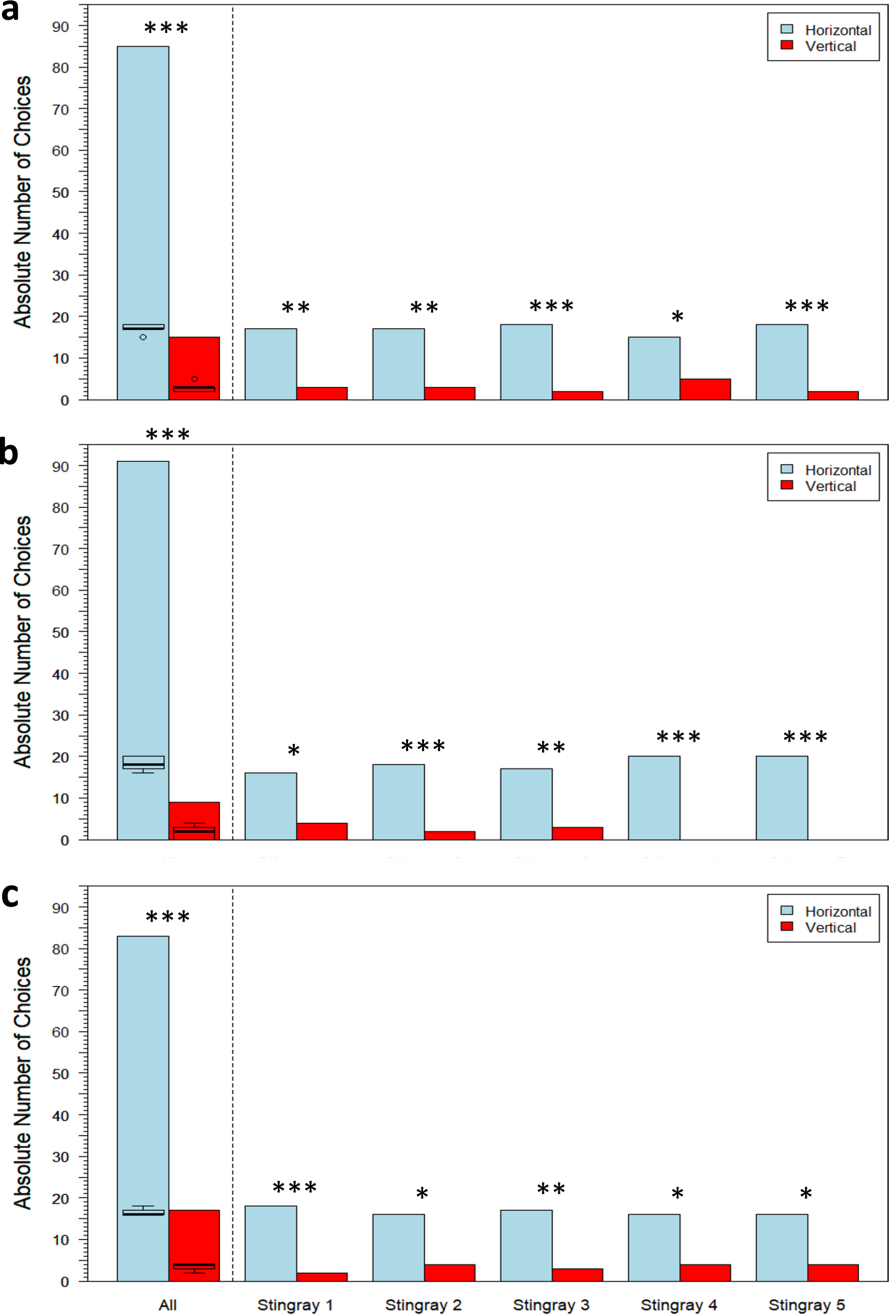
Absolute number of choices between horizontal vs. vertical stimuli for all five stingrays compiled as well as per individual in **a** Transfer 1: black stripe width = 1 cm, five black stripes; **b** Transfer 2: black stripe length = 4 cm, two black stripes; **c** Transfer 3: black stripe width = 8 cm, one black stripe. Boxplots display summary statistics for each stimulus, wherein bold lines indicate medians, color-shaded regions represent interquartile ranges (IQR), dashed bars indicate ranges to ± 1.5 × IQR, and open circles indicate outliers. *p value < 0.05; **p value < 0.01; ***p value < 0.001

Stingrays that were presented with horizontal vs. vertical stimuli achieved LC with a group mean of 9 ± 5 sessions and a median of 9 sessions (Fig. 6c, n = 5). Across three transfer tests, stingrays consistently chose the horizontal stimulus more frequently than the vertical stimulus (Transfer 1: GLMM: *df* = 98, *z* = 6.194, one-sided *p* value < 0.0001; Transfer 2: GLMM: *df* = 98, *z* = 4.355, one-sided *p* value < 0.0001; Transfer 3: GLMM: *df* = 98, *z* = 5.956, one-sided *p* value < 0.0001; Fig. 9). These results showed that discrimination was based on the horizontal and vertical orientation of the lines. Individual results are also displayed in Fig. 9, with the Exact Binomial test used to determine statistical significance (test proportion = 0.5). There were significant differences between average session time and transfer test time in Transfer 1 (Wilcoxon signed-rank test: *V* = 3450, *p* value = 0.001) and Transfer 3 (Wilcoxon signed-rank test: *V* = 4777, *p* value < 0.0001), but not in Transfer 2 (Wilcoxon signed-rank test: *V* = 2595.5, *p* value = 0.81).

### Resolution

Together, stingrays significantly often chose the horizontal stimulus over the vertical stimulus in all transfer tests except Transfer 2 (Transfer 1: *n* = 5, GLMM: *df* = 98, *z* = 2.762, one-sided *p* value = 0.006; Transfer 2: *n* = 5, GLMM: *df* = 98, *z* = 1.79, one-sided *p* value = 0.07; Transfer 3: *n* = 5, GLMM: *df* = 98, *z* = 4.53, one-sided *p* value < 0.0001; Transfer 4: *n* = 5, GLMM: *df* = 98, *z* = 2.182, one-sided *p* value = 0.03; Transfer 5: *n* = 5, GLMM: *df* = 98, *z* = 3.751, one-sided *p* value < 0.001; Transfer 6: *n* = 5, GLMM: *df* = 98, *z* = 2.779, one-sided *p* value = 0.005; Transfer 7: *n* = 3, GLMM: *df* = 58, *z* = 2.832, one-sided *p* value = 0.005; Transfer 8: *n* = 2, GLMM: *df* = 38, *z* = 2.167, one-sided *p* value = 0.03; Table 3). There were significant differences between average session time and transfer test time in all transfer tests (Transfer 1: Wilcoxon signed-rank test: *V* = 4612, *p* value < 0.0001; Transfer 2: Wilcoxon signed-rank test: *V* = 4522, *p* value < 0.0001; Transfer 3: Wilcoxon signed-rank test: *V* = 4360, *p* value < 0.0001; Transfer 4: Wilcoxon signed-rank test: *V* = 4681.5, *p* value < 0.0001; Transfer 5: Wilcoxon signed-rank test: *V* = 4483.5, *p* value < 0.0001; Transfer 6: Wilcoxon signed-rank test: *V* = 4570, *p* value < 0.0001; Transfer 7: Wilcoxon signed-rank test: *V* = 1598.5, *p* value < 0.0001; Transfer 8: Wilcoxon signed-rank test: *V* = 747, *p* value < 0.0001).

**Table 3:** Performance per individual per transfer test and visual acuity calculated per individual

Test (mm)	Stingray 1		Stingray 2		Stingray 3		Stingray 4		Stingray 5	
	*H*	*p*	*H*	*p*	*H*	*p*	*H*	*p*	*H*	*p*
T1 (1.0)	13	0.26	14	0.12	10	1	12	0.50	**15**	0.04
T2 (2.0)	13	0.26	10	1	11	0.82	14	0.12	11	0.82
T3 (10.0)	14	0.12	**19**	< 0.0001	**15**	0.04	**18**	< 0.001	**19**	< 0.0001
T4 (5.0)	10	1	14	0.12	13	0.26	11	0.82	13	0.26
T5 (7.5)	14	0.12	**17**	0.003	11	0.82	**18**	< 0.001	**17**	0.003
T6 (6.5)	10	1	**16**	0.01	11	0.82	**16**	0.01	**17**	0.003
T7 (5.5)	–	––	11	0.82	–	–	**16**	0.01	**15**	0.04
T8 (8.0)	12	0.50	–	–	**17**	0.003	–	–	–	–
CW	–		13		16		11		11	
VA	< 0.13		0.19		0.16		0.23		0.23	

Performance is reported via total choices of horizontal stimulus (*H*), out of 20 total choices, and Exact Binomial *p* value (*p*), given test proportion = 0.5. *H* values in bold are statistically significant scores. Cycle width (CW) is reported in mm (see Eq. 1). Visual acuity (VA) is reported in cycles per degree (cpd)

Due to apparent disagreement between group and individual analyses as well as to variation among individual results, visual acuity was calculated separately for individual animals, ranging from < 0.13 to 0.23 cpd (Table 3).

### Preliminary tests of memory

In the stimulus-presence vs. stimulus-absence experiments, two out of the four stingrays exposed to a 14-day break following LC achieved LC again within three and six sessions after the break, respectively. One stingray scored 80 and 100% in the two sessions immediately following the break but did not achieve LC again within 10 sessions. The fourth, often scored ≥ 70% correct choice following the break but also did not achieve LC again within 10 sessions. The fifth stingray from this group, which had not yet reached LC, officially achieved LC in the first session following the break and maintained ≥ 70% correct choice for three consecutive sessions after the break. This suggests that the discrimination had already been learned before the break.

In the Form and Contrast Experiment B, one stingray achieved LC again within three sessions after a 14-day break. Of the two other stingrays that were exposed to a break before they had achieved LC, one achieved LC four sessions after the break, suggesting that the pause did not interrupt learning. The second individual did not achieve LC in this experiment.

## Discussion

This study describes a series of visual discrimination experiments in *Potamotrygon motoro*. Hypotheses included that stingrays would successfully discriminate (a) stimulus-presence from stimulus-absence, (b) different forms, (c) different overall stimulus contrasts, and (d) horizontal from vertical stimulus orientations. All of these hypotheses were supported, although caution must be observed with regard to form discrimination, as will be discussed. The conclusion of an earlier study regarding color discrimination was potentially corroborated, although the colorful stimuli used in the present study were not controlled for with regard to brightness. This report also details the first visual resolution experiment conducted in *P. motoro* and presents the first evidence of memory retention without reinforcement for this species.

Important limitations of this study are that individual variation between test subjects has a greater influence over results that are obtained from smaller sample sizes and that different trainers conducted each of the visual discrimination experiments described. Both of these conditions were unavoidable, given animal availability and the high investment of time and effort required.

### Visual discrimination

#### Presence vs. absence

To ensure that stingrays were not blind or cognitively impaired, all experimental animals were tested for the ability to discriminate a black form on a white background from a blank white card prior to further experiments. Only the results for the naïve experimental animals are included in this report, as these are not comparable to the results of experienced animals. All individuals achieved LC, so they were considered fit for further experimentation. Across sessions, the average trial time usually decreased, which likely resulted from animals becoming familiar with the experimental procedure and/or the visual task. There was a potential difference in average trial time across sessions between males and females for the eight stingrays presented with the circle stimulus (Fig. 5), so future studies might consider exploring sex differences in behavioral cognition for *P. motoro*. Differences in peripheral sensory input have been documented between the sexes in some rays (e.g. Kempster et al. 2013), and sex differences in cognitive behavior have been documented in other fish, such as guppies (e.g. Lucon-Xiccato and Bisazza 2014). However, there is an unfortunate lack of ecological understanding of *P. motoro*. Based on known differences in parenting roles and sociality in guppies (e.g. Houde 1997; Croft et al. 2004), there is more reason to investigate sex differences in guppies than in these stingrays. Studies to further knowledge of *P. motoro* ecology would aid in understanding the results of any experiments concerning their cognitive behavior.

#### Form, color, and contrast

Two different experimental approaches were used to explore whether *P. motoro* can discriminate form. One group of stingrays (Experiment A) was first trained with two black shapes that were not successfully discriminated and were consequently presented in color. Once the fish learned to discriminate these colorful stimuli, three transfer tests were conducted to determine whether discrimination was based on color/brightness, shape or perhaps both. The second group of stingrays (Experiment B) was trained with two circle stimuli that had different overall contrasts, the design of which was inspired by the species’ dorsal patterning, which is composed of dots with darker, ring-like outlines. Once LC had been achieved, the three stingrays that continued to participate were exposed to two transfer tests, one to confirm that they could discriminate overall contrast and one to investigate whether they had registered information regarding form. Experiment A indicated that stingrays had based discrimination only on color/brightness, while Experiment B indicated that stingrays were able to (a) discriminate contrast and transfer this discrimination to stimuli with unfamiliar form and (b) discriminate a stimulus with the same contrast but a different form from the normally rewarded stimulus. Due to fluctuations in individual performance in the second experiment, it is important to be cautious in interpreting these results, but it would be an obvious mistake at this point to conclude that *P. motoro* cannot discriminate shapes. Parameters to consider in future studies of form discrimination include what shapes should be used in such experiments. Experiment A used a circle versus cross stimulus pair, and as dissimilar as these shapes may seem to the experimenter, it is possible that the rather radial nature of a cross resembles a circle, dependent on distance and visual acuity perhaps. It seems reasonable to conclude that any attempts to investigate form discrimination, also in other species, should prioritize stimulus simplicity in early experiments. In Experiment B, animals were trained with round forms and then presented, in transfer tests, with angular forms (i.e. triangles), possessing the minimum number of corners for two-dimensional shapes. It should be noted that all forms were symmetrical in this study, so asymmetry was not a confounding factor.

#### Stimulus orientation

An ability to discriminate stimulus orientation was investigated by training stingrays with horizontal versus vertical stripes and using three transfer tests to elucidate whether discrimination was truly based on stimulus orientation or on overall stimulus images. Stingrays succeeded in the initial discrimination and continued to significantly often choose the horizontal stimuli during transfer tests, indicating that discrimination was indeed based on stimulus orientation and not on overall images. This sort of ability might be useful in identifying vertical or horizontal obstructions or shelters in the riverine environment or perhaps for recognizing the bodily orientations of other animals.

### Visual resolution and perception

Riggs (1965) distinguishes between four measures of visual acuity (detection, recognition, localization, and resolution), regarding resolution as most critical. This measure is concerned with an animal’s ability to distinguish the elements of an object or stimulus. To evaluate visual resolution in *P. motoro*, the present study included a second transfer test phase in which regular trials still presented stingrays with the striped stimuli from the stimulus orientation experiments. Eight transfer tests were conducted in which stripe widths varied from 1 to 10 mm. The narrowest cycle widths that individuals were able to discriminate were used to calculate visual acuity, which ranged from < 0.13 to 0.23 cpd. Behavioral estimates seem to be much lower than anatomical estimates in elasmobranchs (e.g. Ryan et al. 2017), so these low visual acuity values would be expected if the anatomical estimates of 5.52–6.9 cpd for stingrays in the family Dasyatidae (Garza-Gisholt et al. 2015) are shown to also be representative of Potamotrygonidae.

The crescent shape of the *P. motoro* pupil (Fig. 1) also poses consequences for perception and limits to resolution that should be considered in visual experiments. Murphy and Howland (1991) elaborated on the functional significance of such structures, citing four effects. First, crescent-shaped pupils minimize lenticular spherical aberration by restricting incoming light rays to an equidistant distribution around the lens center; this is in contrast to a circular pupil, which results in a difference between refraction of light rays that pass through the lens periphery and refraction of those passing along the central axis. Second, the presence of the pupillary operculum affects contrast modulation, since an expanding operculum enhances fine details (high spatial frequencies) of a stimulus but reduces the overall stimulus information (low spatial frequencies), including shape. This means the effects of a crescent-shape on contrast modulation and form discrimination may be differentially disadvantageous at certain light levels. Third, Murphy and Howland speculate that the crescent-shaped pupil may function as a focus indicator for organisms lacking a fovea; if the lens focuses in front of an object, points of light reflected off the object appear as ‘U’ shapes, while focusing behind the object results in an inverted ‘U’. Lastly, as a crescent-shaped pupil is constricted, depth of field is reduced while the theoretical limit to resolution is increased, effects which are opposite in a constricting circular pupil. This implies that, at higher light levels, a stingray eye should probably receive images that are more detailed but only over reduced distances. In an attempt to avoid the influence of elevated light levels on results, the present study limited light intensity in the experimental rooms to 320 lx.

In support of the low acuity values found by this study, the environmental and ecological demands on vision for *Potamotrygon motoro* do not seem to require especially high visual acuity, given the often-elevated sediment loads in riverine waters (e.g. Ríos-Villamizar et al. 2014; Costa et al. 2011), an apparent lack of predators, aside from humans (Charvet-Almeide et al. 2002), and often tactile foraging behaviors (Garrone-Neto and Sazima 2009), though *P. motoro*’s habitat use and foraging behavior do transition with age (Garrone-Neto and Sazima 2009). The group of *P. motoro* used in this resolution experiment were already sub-adult, and normally, visual acuity actually improves as fish develop (e.g. Pankhurst et al. 1993). However, larval rainbow trout (*Oncorhynchus mykiss*) behaviorally demonstrated an acuity of 40 cpd at 10 days after hatching which exponentially decreased to 6.5 cpd at 15 days and 1.4 at 75 days (Carvalho et al. 2004). Adult tuna achieved visual acuity scores < 0.20 cpd (Nakamura 1968), similar to *P. motoro*.

Studies on larval fish have shown that there is a mismatch between theoretical and behavioral spatial acuity, potentially explainable by the myopia of the larval fish eye (Pankhurst et al. 1993), but, according to Browman et al. 1990, behavioral estimates are probably more accurate than anatomical estimates of visual acuity anyway, since behavior is influenced by a broad range of neurological factors. For example, optokinetic response experiments with larval zebrafish (*Danio rerio*) resulted in an acuity of 0.16 cpd while the theoretical limit based on anatomy was 0.24 cpd (Haug et al. 2010).

It is unfortunately difficult to compare studies on visual resolution between species, as well as within species, due to differences in experimental approaches, conditions, and animal ages. Various measurements of visual acuity have been derived from optokinetic or optomotor responses, as in the aforementioned rainbow trout studies (Carvalho et al. 2004), or from reaction distances and morphological measurements, as was done in the case of seahorses (e.g. Lee and O’Brien 2011), or from thresholds of stimulus resolution, as with tuna (Nakamura 1968) and the present study. The present study used similar stimuli and the same formula as Nakamura (1968), so those results for tuna are probably the most comparable to our findings for *P. motoro*. However, Nakamura (1968) used a projector to display the visual stimuli and was therefore additionally able to compare visual resolutions at different stimulus luminance levels between the two tuna species. He found that resolution was comparable at lower luminance but that skipjack tuna (*Katsuwonus pelamis*) had better resolution abilities at higher values of luminance.

The importance of light conditions regarding a species’ visual apparatus cannot be understated (Murphy and Howland 1991), and neither can the choice of visual stimulus. Honeybees (Srinivasan and Lehrer 1988) and triggerfish (*Rhinecanthus aculeatus*; Champ et al. 2014), for example, achieved lower scores of visual acuity when presented with radial stimuli instead of linear stimuli. In the previously discussed Form, Color, and Contrast experiments, the present study shows that stingrays seem to pay attention to information about color/brightness over information about shape. Interestingly, Schluessel and Ober (2018) were able to conclude that *Potamotrygon motoro* prefers directional cues to visual stimulus cards or landmark cues in solving a navigational task. Perhaps it is worth considering that such stimulus preferences could play a role in participation/performance differences between visual discrimination tasks. The experimental design and focus of the present experiment was of course different from that of Schluessel and Ober (2018), but it shows that stingrays participating in stimulus orientation experiments performed more consistently and usually at 100% correct choice as compared to the more fluctuating performance curves of stingrays in other experiments (Fig. 3). Whether or not horizontal vs. vertical stripes can somehow be considered ‘directional’ cues is, however, not clear, and whether differences in performance curves could indicate preferences for certain stimuli is subject to confounding information.

Lastly, it is difficult to determine when an animal has made a stimulus choice. In the present study, each stingray may have made its decision at a different distance from the stimulus wall, but since these distances could not be objectively determined with certainty, a standard distance was used to calculate visual acuity for all individuals. Relatedly, it would be worthwhile for future studies to explore whether the angle of the visual stimulus relative to the stingray has an effect on experimental results. There is no information about photoreceptor or ganglion cell distribution in the eye of *P. motoro* to date, let alone correlations between the two, but a study comparing closely related stingray species in the family Dasyatidae found that differences in the distribution of retinal neurons seem related to ecology (Garza-Gisholt et al 2015). Future studies with *P. motoro* should pair behavioral experiments with anatomical investigations and consider exploring whether ontogeny correlates with visual acuity, as in the case of the variable threefin, *Forsterygion varium* (Pankhurst et al. 1993).

### Preliminary tests of memory

An additional pilot investigation into the memory capacity of *Potamotrygon motoro* was conducted, in which a 14-day break in reinforcement was included during either the stimulus-presence vs. stimulus-absence experiments or the training phase of Form and Contrast Experiment B. Having already achieved LC before the break, two out of four stingrays in the stimulus-presence vs. stimulus-absence experiments were able to quickly (i.e. in less than seven sessions) achieve LC again following the break in reinforcement, indicating that they remembered the task. A fifth stingray was given a break after just two consecutive sessions of ≥ 70% correct choice but officially achieved LC in the first session following the break and maintained ≥ 70% correct choice for two more sessions. This suggests the stingray had already learned the association before LC had been achieved and, furthermore, remembered the association after the break.

Three stingrays in Form and Contrast Experiment B were also exposed to a 14-day break. Having achieved LC prior, one individual achieved LC again after the break, which indicates that memory was not impeded by the use of different, black and white stimuli. The other two stingrays were given a break before they had achieved LC, after only 7 or 14 sessions, to see how soon they would achieve LC thereafter. The individual given a break after 14 sessions achieved LC just four sessions after the break, so the break did not impede the learning process in this individual. The second individual did not achieve LC within 51 sessions, after which the experiment was terminated for that individual. It is, however, highly unlikely that a break in training causes long-term impacts on cognitive ability, so this outcome is probably explainable by individual variation.

## References

[CR1] Ali MA, Anctil M (1974). Retinas of the electric ray (*Narcine brasiliensis*) and the freshwater stingray (P*otamotrygon motoro)*. Vis Res.

[CR2] Bedore CN, Loew ER, Frank TM, Hueter RE, McComb DM, Kajiura SM (2013). A physiological analysis of colour vision in batoid elasmobranchs. J Comp Physiol A.

[CR3] Browman HI, Gordon WC, Evans BI, O’Brien WJ (1990). Correlation between histological and behavioral measures of visual acuity in a zooplanktivorous fish, the white crappie (*Pomoxis annularis*). Brain Behav Evol.

[CR4] Carvalho PS, Noltie DB, Tillitt DE (2004). Biochemical, histological and behavioral aspects of visual function during early development of rainbow trout. J Fish Biol.

[CR5] Champ C, Wallis G, Vorobyev M, Siebeck U, Marshall J (2014). Visual acuity in a species of coral reef fish: *Rhinecanthus aculeatus*. Brain Behav Evol.

[CR6] Charvet-Almeida P, Araújo MLG, Rosa R (2002). Neotropical freshwater stingrays: diversity and conservation status. Shark News.

[CR7] Christofzik N (2016) Visuelle Wahrnehmung und Diskriminierung numerischer Informationen beim Pfauenaugenstechrochen (*Potamotrygon motoro*). University of Bonn, Bachelor thesis

[CR8] Costa MP, Telmer K, Novo EM (2011) Spatial and Temporal Variability of Light Attenuation in the Amazonian Waters. Conference Proceedings found athttps://citeseerx.ist.psu.edu/viewdoc/download?doi=10.1.1.368.1799&rep=rep1&type=pdf

[CR9] Croft DP, Krause J, James R (2004). Social networks in the guppy (*Poecilia reticulata*). Proc R Soc B.

[CR10] Daniel MMM, Schluessel V (2020). Serial reversal learning in freshwater stingrays (*Potamotrygon motoro*). Anim Cogn.

[CR11] Fuss T, Bleckmann H, Schluessel V (2014). Visual discrimination abilities in the gray bamboo shark (*Chiloscyllium griseum*). Zool.

[CR12] Garamszegi LZ (2015). A simple statistical guide for the analysis of behavior when data are constrained due to practical or ethical reasons. Anim Behav.

[CR13] Garrone Neto D, Uieda VS (2012). Activity and habitat use of two species of stingrays (Myliobatiformes: Potamotrygonidae) in the upper Paraná River basin, Southeastern Brazil. Neotrop Ichthyol.

[CR14] Garrone-Neto D, Sazima I (2009). Stirring, charging, and picking: hunting tactics of potamotrygonid rays in the upper Paraná River. Neotrop Ichthyol.

[CR15] Garza-Gisholt E, Kempster RM, Hart NS, Collin SP (2015). Visual specializations in five sympatric species of stingrays from the family Dasyatidae. Brain Behav Evol.

[CR16] Gustafsson OSE, Ekström P, Kröger RHH (2012). Sturgeons, sharks, and rays have multifocal crystalline lenses and similar lens suspension apparatuses. J Morphol.

[CR17] Hart NS, Lisney TJ, Marshall NJ, Collin SP (2004). Multiple cone visual pigments and the potential for trichromatic colour vision in two species of elasmobranch. J Exp Biol.

[CR19] Haug MF, Biehlmaier O, Mueller KP, Neuhauss SC (2010). Visual acutiy in larval zebra fish: behavior and histology. Front Zool.

[CR20] Houde AE (1997). Sex, color, and mate choice in guppies.

[CR21] Kempster RM, Garza-Gisholt E, Egeberg CA, Hart NS, O'Shea O, Collin SP (2013). Sexual dimorphism of the electrosensory system: a quantitative analysis of nerve axons in the dorsal anterior lateral line nerve of the blue-spotted fantail stingray (*Taeniura lymma*). Brain Behav Evol.

[CR22] Leal M, Powell BJ (2012). Behavioural flexibility and problem-solving in a tropical lizard. Biol Lett.

[CR23] Lee HR, O’Brien KMB (2011). Morphological and behavioral limit of visual resolution in temperate (*Hippocampus abdominalis*) and tropical (*Hippocampus taeniopterus*) seahorses. Vis Neurosci.

[CR24] Lucon-Xiccato T, Bisazza A (2014). Discrimination reversal learning reveals greater female behavioral flexibility in guppies. Biol Lett.

[CR25] Murphy CJ, Howland HC (1991). The functional significance of crescent-shaped pupils and multiple pupillary apertures. J Exp Zool.

[CR26] Nakamura EL (1968). Visual acuity of two tunas, *Katsuwonus pelamis* and *Euthynnus affinis*. Copeia.

[CR27] Niederbremer C (2019) Diskriminierung kleiner Mengenverhältnisse beim Pfauenaugen-stechrochen (*Potamotrygon motoro*). University of Bonn, Bachelor thesis

[CR28] Oliveira AT, Araújo MLG, Lemos JRG, Santos MQC, Pantoja-Lima J, Aride PHR, Tavares-Dias M, Marcon JL (2017). Ecophysiological interactions and water-related physicochemical parameters among freshwater stingrays. Braz J Biol.

[CR29] Pankhurst PM, Pankhurst NW, Montgomery JC (1993). Comparison of behavioural and morphological measures of visual acuity during ontogeny in a teleost fish, *Forsterygion varium*, Tripterygiidae (Forster, 1801). Brain Behav Evol.

[CR30] Parker AN, Fritsches KA, Newport C, Wallis G, Siebeck UE (2017). Comparison of functional and anatomical estimations of visual acuity in two species of coral reef fish. J Exp Biol.

[CR31] Riggs LA, Graham CH (1965). Visual acuity. Vision and visual perception.

[CR32] Ríos-Villamizar EA, Piedade MTF, Da Costa JG, Adeney JM, Junk WJ (2014). Chemistry of different Amazonian water types for river classification: a preliminary review. WIT Trans Ecol Environ.

[CR33] Rosa RS (1985) A systematic revision of the South American freshwater stingrays (Chondrichthyes: Potamotrygonidae). Thesis. College of William and Mary, Williamsburg, Virginia 523 p

[CR34] Ryan LA, Hemmi JM, Collin SP, Hart NS (2017). Electrophysiological measures of temporal resolution, contrast sensitivity and spatial resolving power in sharks. J Comp Physiol A.

[CR35] Schluessel V (2015). Who would have thought that ‘Jaws’ also has brains? Cognitive functions in Elasmobranchs. Anim Cogn.

[CR36] Schluessel V, Bleckmann H (2005). Spatial memory and orientation strategies in the elasmobranch *Potamotrygon motoro*. J Comp Physiol A.

[CR37] Schluessel V, Ober C (2018). How to get out of a maze? Stingrays (*Potamotrygon motoro*) use directional over landmark information when provided with both in a spatial task. Evol Ecol Res.

[CR38] Schluessel V, Rick IP, Plischke K (2014). No rainbow for grey bamboo sharks: evidence for theabsence of colour vision in sharks from behavioural discrimination experiments. J Comp Physiol A.

[CR39] Schluessel V, Herzog H, Scherpenstein M (2015). Seeing the forest before the trees–spatial orientation in freshwater stingrays (*Potamotrygon motoro*) in a hole-board task. Behav Proc.

[CR40] Seifert FD (2017) Farbwahrnehmung beim Pfauenaugenstechrochen (*Potamotrygon motoro*). University of Bonn, Bachelor thesis

[CR41] Sivak JG (1991). Elasmobranch Visual Optics. J Exp Zool.

[CR42] Srinivasan MV, Lehrer M (1988). Spatial acuity of honeybee vision and its spectral properties. J Comp Physiol A.

[CR43] Theiss SM, Lisney TJ, Collin SP, Hart NS (2007). Colour vision and visual ecology of the blue-spotted maskray, Dasyatis kuhlii Muller & Henle, 1814. J Comp Physiol A.

[CR44] Van-Eyk SM, Siebeck UE, Champ CM, Marshall J, Hart NS (2011). Behavioural evidence for colour vision in an elasmobranch. J Exp Biol.

